# Response Properties of Human Amygdala Subregions: Evidence Based on Functional MRI Combined with Probabilistic Anatomical Maps

**DOI:** 10.1371/journal.pone.0000307

**Published:** 2007-03-21

**Authors:** Tonio Ball, Benjamin Rahm, Simon B. Eickhoff, Andreas Schulze-Bonhage, Oliver Speck, Isabella Mutschler

**Affiliations:** 1 Epilepsy Center, University Hospital Freiburg, Freiburg, Germany; 2 Heidelberg Academy of Science and Humanities, Heidelberg, Germany; 3 Freiburg Brain Imaging, University Hospital Freiburg, Freiburg, Germany; 4 Bernstein Center for Computational Neuroscience Freiburg, Freiburg, Germany; 5 Departement of Cognitive Psychology and Methodology, University of Basel, Basel, Switzerland; 6 Institute of Medical Psychology, University of Frankfurt, Frankfurt, Germany; 7 Institute for Medicine, Research Center Jülich, Jülich, Germany; 8 C&O Institute for Brain Research, University of Düsseldorf, Düsseldorf, Germany; 9 Departement of Biomedical Magnetic Resonance, Institute for Experimental Physics, University of Magdeburg, Magdeburg, Germany; University of Minnesota, United States of America

## Abstract

The human amygdala is thought to play a pivotal role in the processing of emotionally significant sensory information. The major subdivisions of the human amygdala—the laterobasal group (LB), the superficial group (SF), and the centromedial group (CM)—have been anatomically delineated, but the functional response properties of these amygdala subregions in humans are still unclear. We combined functional MRI with cyto-architectonically defined probabilistic maps to analyze the response characteristics of amygdala subregions in subjects presented with auditory stimuli. We found positive auditory stimulation-related signal changes predominantly in probabilistically defined LB, and negative responses predominantly in SF and CM. In the left amygdala, mean response magnitude in the core area of LB with 90–100% assignment probability was significantly larger than in the core areas of SF and CM. These differences were observed for pleasant and unpleasant stimuli. Our findings reveal that the probabilistically defined anatomical subregions of the human amygdala show distinctive fMRI response patterns. The stronger auditory responses in LB as compared with SF and CM may reflect a predominance of auditory inputs to human LB, similar to many animal species in which the majority of sensory, including auditory, afferents project to this subdivision of the amygdala. Our study indicates that the intrinsic functional differentiation of the human amygdala may be probed using fMRI combined with probabilistic anatomical maps.

## Introduction

The challenge of unraveling the function of the human amygdala is attracting great interest [1]–[5]. This is reflected in a broad range of neuroimaging studies that includes investigations of emotional processing of chemosensory information [6]–[8], visual stimuli such as facial emotional expression [9]–[11], and auditory stimuli including communication sounds [12], [13] and music [14], [15]. In addition to the desire to understand the neuronal basis of human emotion, an important motivation behind these studies is to understand the role of the amygdala in neuro-psychiatric diseases such as depression, anxiety disorders, and antisocial personality disorder [16]–[19]. Studies in many mammalian species [2], [20]–[25] including humans [26] have firmly established that the amygdala is not a single homogenous structure but that it is composed of several anatomical groups of subnuclei. In animal research, investigation of the intrinsic amygdaloid network and information flow plays a crucial role and is generally thought to be a key issue for elucidating amygdala function [27]. Similarly, greater understanding of amygdala function in humans may be achieved by differentiating response properties of human amygdala subregions.

There is however a lack of such data in humans, the major reason for which is that current structural brain scans do not enable the differentiation of individual subregions of the human amygdala [26]. This problem is compounded by the fact that the exact location of the amygdala subregions varies between individuals and that standard atlas systems do not provide information about this inter-individual anatomical variability. We therefore combined functional magnetic resonance imaging (fMRI) with probabilistic anatomical maps [26] based on histological analysis of ten human post-mortem brains. The advantage of such probabilistic maps is that they provide information about the location and inter-individual variability of brain areas in standard reference space. This allows assignment of activation sites to micro-anatomically defined brain regions in a probabilistic fashion [28], even if these brain regions are not discernible in structural brain images.

The major amygdala subdivisions and their assumed function as established in animal research are as follows: There is evidence in mammalian species including monkeys that the majority of subcortical and cortical inputs converge in the laterobasal group [2], [20]–[25]. This structure is believed to play a crucial role in assigning emotional value to sensory stimuli [29]. The superficial (cortical) part of the amygdala is a neighboring structure of the laterobasal group. Its function has been investigated less thoroughly. The acquisition of a conditioned defensive response in normal rats has however been shown to be associated with increased metabolic activity in the area of the superficial group, suggesting an involvement of this subregion in affective processing [30]. The centromedial group receives convergent information from several other amygdaloid regions and sends efferents to various subcortical structures, generating behavioural responses such as modulation of autonomic activity [21], [27].

Similarly, the human amygdala is not a homogeneous anatomical structure. Based on differences in cyto-, myelo-, and chemoarchitecture [26], it can be differentiated into the laterobasal amygdaloid group (LB), the centromedial group (CM), and the superficial group (SF) [26], [31]. LB comprises the lateral, basolateral, basomedial and paralaminar nuclei, CM the central and the medial nuclei, and SF includes the anterior amygdaloid area, the ventral and posterior cortical nuclei [26]. Results from previous neuroimaging studies [9]–[11] suggest functional differences in the human amygdala region similar to those of the animal amygdala, but response differences between LB, SF, and CM, were not directly assessed in these studies. The specific functional response properties of these major subdivisions of the human amygdala are therefore unclear.

On this background, the principle aim of our study was to determine the response properties of probabilistically defined LB, SF, and CM of the human amygdala during processing of emotionally significant sensory information. To investigate this, piano melodies were presented to fourteen healthy subjects during acquisition of blood oxygenation level dependent (BOLD) contrast sensitive functional MR images in a 3T scanner. The distribution of BOLD signal changes was then analyzed using probabilistic maps of the three anatomical amygdala subregions [26].

## Material and Methods

### Subjects

Fourteen subjects (9 females, 5 males, mean age = 23.7 years, range = 20–34 years) without professional musical education participated in this study after giving written informed consent. The study was approved by the ethics committee of the University of Freiburg, Germany. All participants had no history of psychiatric or neurological disease or hearing disorders. Subjects were right-handed according to the Edinburgh handedness questionnaire [32]: mean = 82.6%, range = 75–100%.

### Stimuli

For auditory stimulation, 10 piano pieces, each of 24 second duration, were presented. The musical stimuli were selected in cooperation with a professional musician and chosen from the romantic period. All pieces were examples of major-minor tonal music. The tempo and harmony of the 10 selected piano melodies (see Table 1) were varied, creating four versions of each tune: consonant-fast (CF), consonant-slow (CS), dissonant-fast (DF), and dissonant-slow (DS). Tempi for the fast and slow versions were 156 and 84 beats per minute, respectively. These values were selected because they represented the fastest/slowest tempi that still sounded natural to one professional musician and five non-musicians. The consonant stimuli were the original tunes, whereas the dissonant stimuli were electronically manipulated counterparts of the original tunes, created by shifting the melody of the original excerpt, but not the accompanying chords, by a half tone below the original pitch. Thus, the dissonant and consonant, and the fast and slow versions of a tune had the same rhythmic structure and the identical melodic contour. All stimuli were processed using Cubase VST/32 R.5 (Steinberg) as software. Finally, all sound files were transformed into wave files for stimulation in the scanner (using WaveLab, Steinberg).[Table pone-0000307-t002]

**Table 1:** Composer, opus, and interpreter of the 24 second piano music excerpts used in the fMRI experiment.

Composer	Opus	Interpreter
Schubert	Four Impromptus, D.935, Op.posth.142; No.1 in F	J.E. Dery
Mendelssohn	Book 5, Op.62; 6.Frühlingslied in A	C. Meesangnin
Tchaikovsky	Album pour enfants, Op.39; 5.March of the Wooden Soldiers	M. Knezevic
Chopin	Waltz in Db, Op.69, No.1 (‘L'adieu’)	P.M.P. Blondel
Mozart	Piano Sonata in C, K.330; 2. Andante cantabile	R. Ungar
Liszt	Valse Impromptue	G. Giulimondi
Chopin	Mazurka in A-, Op.67, No.4	R. Lubetsky
Haydn	Keyboard Sonata in G, Hob.XVI:27, Op.14, No.1 (No.42)	T. Leen
Tchaikovsky	12 morceaux, difficulté moyen, Op.40; 6.Song Without Words	M. Knezevic
Schumann	Kinderszenen, Op.15; 2.Kuriose Geschichte	G. Giulimondi

We took care to select stimuli that elicit significant emotional reactions (see results section). In view of the ongoing controversy about whether the human amygdala responds to pleasant and unpleasant stimuli in a similar [33], [34] or rather in a different manner [15] we sought to select stimuli that evoke either pleasant or unpleasant emotional reactions. A further point guiding our stimulus selection was that we aimed to use relatively unknown piano melodies, because several studies indicate that the response of the human amygdala to emotional stimuli diminishes with repeated presentations [35]–[38], that is, with increasing familiarity. Consequently, we chose relatively unknown musical excerpts. We assessed familiarity by subjects' ratings immediately after scanning (for details, see the end of the following paragraph).

### Experimental design

The 4 variations of the 10 piano tunes were presented during the fMRI experiment in a random order, using in-house developed presentation software, via magnetic resonance compatible headphones (NordicNeuroLab, Norway). Subjects viewed a fixation cross during the experiment and were instructed to listen attentively to the music and to avoid any overt movement. Each melody was preceded by a written instruction presented on the screen (‘music starts’), and each melody lasted 24 seconds. Each melody was followed by a period of 36 seconds, during which no music was presented. Within this period, participants were asked to rate the tune on a 7-point self-assessment scale along the dimensions valence (ranging from −3 = very unpleasant to 3 = very pleasant) and arousal (ranging from −3 = very calming to 3 = very arousing). Each of the two rating periods lasted 6 seconds. Subject conveyed their decision by using a magnetic resonance compatible mouse which allowed them to move a white box on the visually presented scale leftwards or rightwards by pressing the corresponding mouse button with their right hand. There were 40 runs (each consisting of melody presentation and evaluation). Total scanning time was 42 minutes. After scanning subjects were asked to rate the familiarity (ranging from 0 = not familiar; to 3 = familiar). Subjects gave two global familiarity ratings, one for the pleasant and one for the unpleasant melodies.

### Magnetic Resonance Imaging

Functional and structural images were acquired on a 3 T scanner (Siemens Magnetom Trio, Erlangen, Germany). Structural T1-weighted images were obtained using a MPRAGE sequence (resolution: 1mm isotropic, matrix: 256*256*160, TR: 2200 ms, TI: 1000 ms, 12° flip angle). Functional images were acquired using a multislice gradient echo planar imaging method (EPI). Each volume consisted of 44 sagittal slices (resolution: 3 mm isotropic, matrix: 64*64, FOV 192 mm*192 mm, TR 3000 ms, TE 30 ms, 90° flip angle). The sagittal slice orientation resulted in significantly lower acoustic noise generated by the imaging gradients, enabling a better stimulus perception. In addition, this orientation in combination with the thin slice thickness reduced the signal loss in the amygdala region to give more reliable detection of activation.

**Figure 1:**
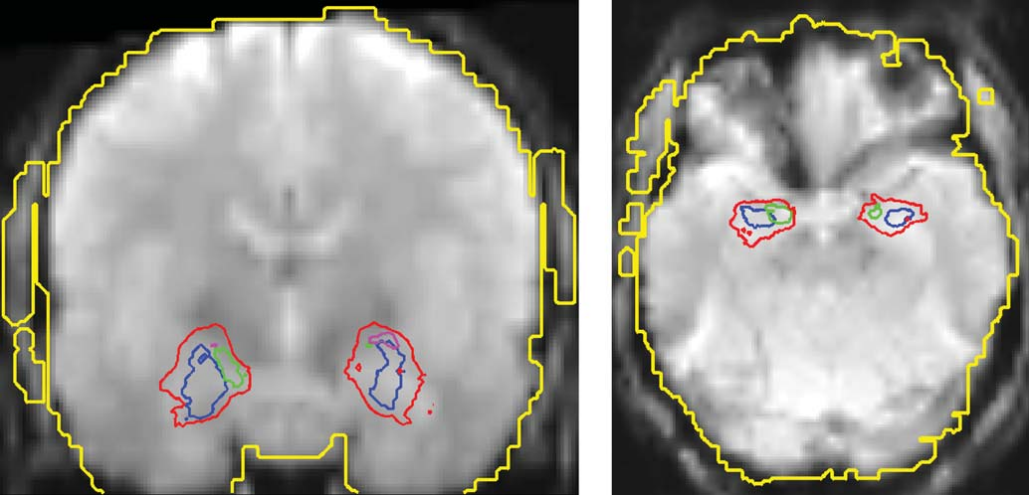
EPI data quality. A representative example of raw EPI (echoplanar imaging) data normalized to MNI space is shown in a coronar (MNI y = −7 mm) and axial (MNI z = −20 mm) section. The axial section corresponds approx. to the sections shown in [51]. The example represents the average across all EPI volumes acquired during the experimental session of one subject. The outline of the amygdala is shown (red line), enclosing the area with at least 50% probability of belonging to the amygdala (according to the probabilistic maps from [26]). The extent of the laterobasal (LB) group of the amygdala (LB, > = 80% probability) is shown in blue, the superficial group (SF) in green, and the centromedial group (CM) in magenta. In addition, the outline of the segmentation mask enclosing the area with sufficient signal for application of point spread function (PSF) based EPI distortion correction (see reference [39] for further details) is shown in yellow. In all subjects, the whole analyzed extent of the amygdala was within the segmentation mask and therefore distortion correction was possible in this region. Good EPI signal quality in the amygdala region was achieved in all subjects investigated.

An accurate registration of the functional and structural images was enabled by correction of the EPI data for geometric distortions [39]. The distortion field was derived from the local point spread function (PSF) in each voxel as determined in a one minute reference scan. Prior to distortion correction, the data were motion corrected by image realignment with the reference scan. Motion and distortion correction were performed online during the reconstruction process. The applicability of the PSF-based image correction to the amygdala region critically rests on a sufficiently strong signal that might be compromised by local signal drop out. We therefore verified that the whole analyzed extent of the amygdala had good EPI signal quality and that distortion correction was possible in this region (Fig. 1).

### Functional Data analysis

The results of the subjects' valence and arousal ratings were analyzed by two-way repeated measurements ANOVA, using Matlab (Version 7.0.4, the Mathworks, USA). Spatial preprocessing and statistical analyses of the functional MR data at the individual level were preformed with SPM5 (Wellcome Department of Cognitive Neurology, London, UK). All functional images were normalized into standard stereotaxic space of the Montreal Neurological Institute (MNI) template and smoothed using a 6 mm full-width-at-half-maximum (FWHM) Gaussian kernel. The timing information of the four music conditions and of the evaluation periods were each modeled with a box-car function convolved with a canonical hemodynamic response function. A high-pass filter with a cut-off of 1/128 Hz was applied before the parameter estimation according to the general linear model used by SPM5. Contrast images of music presentation>implicit baseline (i.e. time periods during which subjects passively viewed the fixation cross without any task and without music presentation) were calculated for all subjects. For each music condition, the BOLD percentage signal change (PSC) was then extracted (using in-house software) for all voxels which were assigned with a probability of least 50% to one of the amygdala subregions LB, CM, or SF (separately for right and left amygdala), using the probabilistic amygdala maps of Amunts and colleagues [26] (the probabilistic anatomical maps can be freely accessed through www.fz-juelich.de/ime/spm_anatomy_toolbox, mirrored on www.bic.mni.mcgill.ca/cytoarchitectonics). Voxels (having 3 mm isotropic size in the functional data) were assigned according to the probability at the voxel center.

Voxelwise between-subjects random effects were statistically evaluated in two ways: (1) based on the mean responses across all four music conditions, and (2) for consonant and dissonant music conditions (collapsing slow and fast conditions), separately. In many of the analyzed voxels, the null hypothesis that the PSC data (both for the mean and consonant/dissonant case) were normally distributed with unspecified mean and variance had to be rejected (p<0.001, 2-sided Bera-Jarque test of composite normality). Therefore nonparametric tests were used for further analysis.

For the random effect analysis, a sign test was used to evaluate the hypothesis that the PSC data (i.e. all 14 values per voxel) came from a distribution with zero median. The significance level for this analysis was p<0.0026, corresponding to a significance of p<0.05 for the whole amygdala (corrected for multiple comparisons based on the number of analyzed resolution elements). To determine the activated/deactivated volume in the amygdala subregions, the voxels with significant effects were up-sampled to 1mm isotropic resolution and the number of resulting voxels within LB, SF, and CM was determined.

Further, histograms with 30 equally spaced bins across the whole range of the voxelwise PSC data were computed, and we determined, separately for LB, SF, and CM, the lateralization index (LI), defined as (Rn-Ln)/(Rn+Ln))*100, with Rn and Ln being the number of voxels in the right and left amygdala for a given PSC bin.

**Figure 2:**
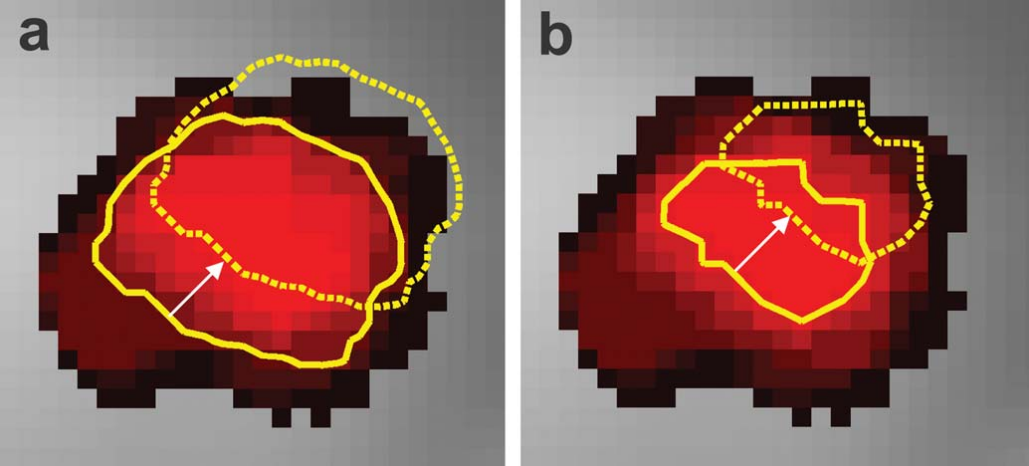
Simulated effect of probabilistic region of interest (ROI) definition on robustness against localization errors. In (a) and (b), axial sections through the laterobasal part (LB) of the left amygdala are shown at z = −29 (MNI coordinate system). Probabilities to belong to LB are color coded with black = 10% and pure red = 100%. In (a), the probabilistic ROI is defined as the area with> = 50% probability to belong to LB (solid yellow line). As a consequence of a simulated localization error which consists of a linear shift of approximately 4 mm (indicated by the white arrow) the true measured volume is localized as indicated by the dashed yellow line, containing voxels with little or no probability to belong to LB. In contrast, when restricting the ROI to voxels with 90% or 100% LB probability (b) and given an equally large localization error, the true measured volume is still entirely located within the area of LB. In this way, the robustness against localization errors could be increased, counteracting potential localization errors while still maintaining excellent anatomical specificity.

In addition to the voxelwise analysis, we also performed region of interest (ROI) analyses for six areas of interest (right and left LB, SF, and CM). In order to increase the robustness of our results against spatial errors, we restricted the ROI to the core of the amygdala subregions with> = 90% assignment probability. As illustrated in Fig. 2, in this way the localisatory assignment is made more robust against spatial localization errors as compared to less strictly defined ROI (e.g. the area with> = 50% assignment probability).

For each of the LB, SF, and CM ROIs, the mean PSC of all voxels (i.e. all voxels with at least 90% probability to belong to a given area) was determined for each of the 14 subjects. The resulting data were normally distributed (p>0.2, Bera-Jarque test). Differences in ROI data between amygdala subdivisions were assessed using a Students t-test for all combinations of the 3 areas of interest, both for the right and left amygdala. As with the voxelwise analysis, the ROI analysis was carried out both for the mean effect across the four music conditions and for the consonant and dissonant music conditions, individually.

## Results

Mean values (+/−standard error of the mean) for valence ratings of DS, DF, CS, and CF stimuli were −1.05 (+/−0.12), −0.92 (+/−0.12), 0.87 (+/−0.12), and 1.06 (+/−0.12). The corresponding results for arousal ratings were −0.86 (+/−0.12), 0.01 (+/−0.13), −0.27 (+/−0.12), and 1.04 (+/−0.12). A two-way repeated measurements ANOVA of the effects of harmony and tempo on valence and arousal ratings showed a significant main effect of harmony on valence ratings and of both harmony and tempo on arousal ratings (p<0.0001).

After scanning, subjects were asked to rate globally the familiarity (ranging from 0 = not familiar; to 3 = familiar) of the pleasant and for the unpleasant music. Familiarity ratings for the pleasant and unpleasant melodies were identical in all cases. The mean familiarity ratings both were 0.72 (SD = 0.87), indicating that musical pieces were on average only slightly familiar to the subjects.

**Figure 3:**
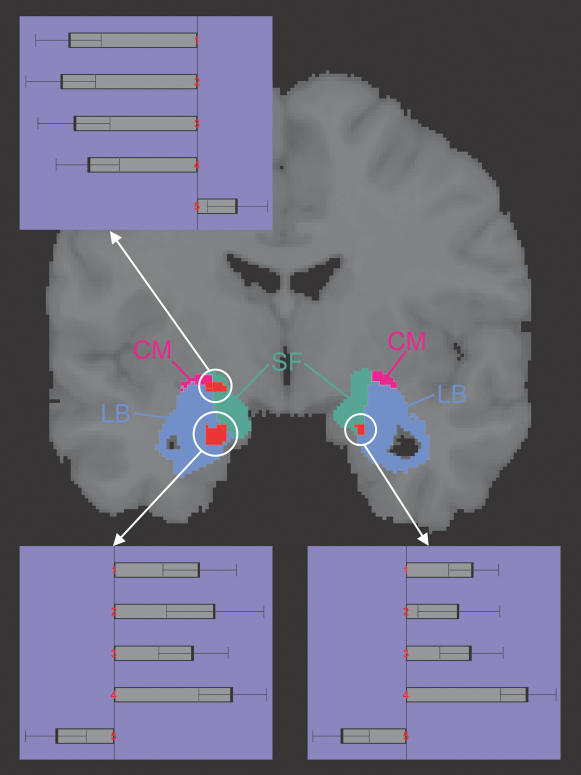
Music-related amygdala responses. The extent of the amygdala subregions LB, SF, and CM defined by maximum-probability maps (MPMs, [28]) are rendered in blue, green, and magenta, respectively, on a coronar section. Regions with significant (p<0.05, corrected for multiple comparisons in the search volume) music-related responses are shown in red. The BOLD percentage signal change (PSC) of each of the three activation sites in the four music conditions (1: dissonant slow, 2: consonant slow, 3: dissonant fast, 4: consonant fast) is presented as mean and standard error. The fifth PSC value refers to the time period during which subjects evaluated the emotional effect evoked by the preceding musical piece, compared with rest. In the left amygdala, a cluster with positive responses to all music conditions was mainly located in LB and a cluster with negative responses was mainly found in SF and CM (see also Tab. 2). A smaller cluster with consistently positive responses was also found in the right amygdala approximately in the mirror position to the positively responding left cluster.

**Figure 4:**
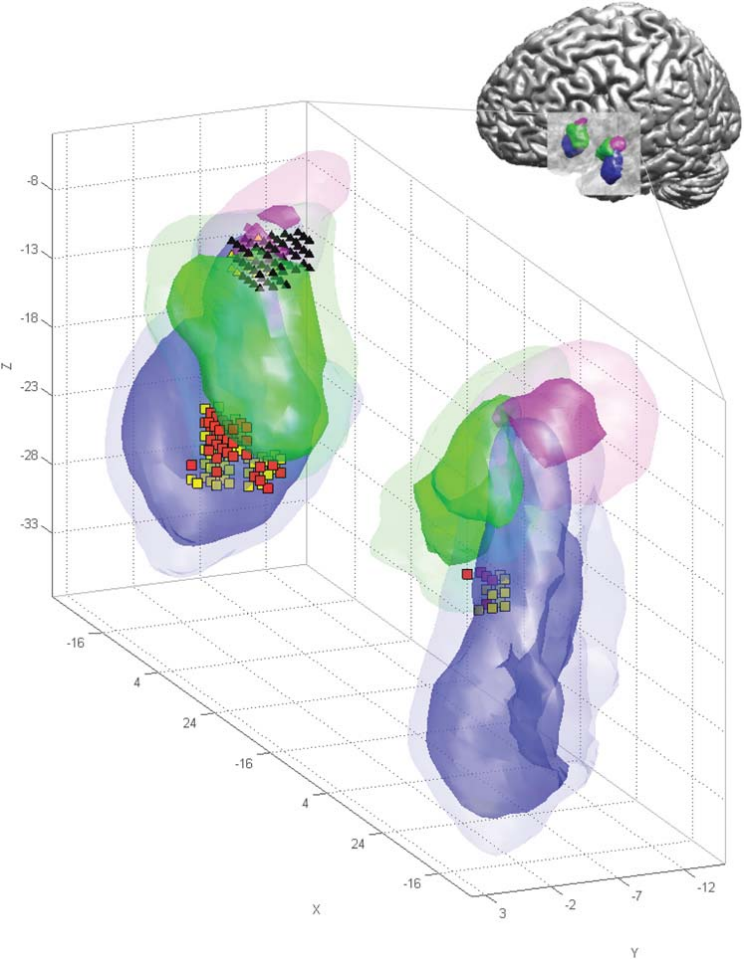
Human amygdala responses to music in probabilistically defined anatomical regions. The volumes with at least 50% and 80% probability to belong to the three major amygdala subregions: LB = laterobasal group (blue), SF = superficial group (green), and CM = centromedial group (magenta) are rendered with high (for 50% probability) and low (for 80% probability) transparency. Voxels with a significant increase in BOLD signal to music presentation are displayed as red or yellow squares (p<0.05, corrected for multiple comparisons, see material and methods for further details). Voxels with a significant signal decrease are displayed as black or yellow triangles. Anatomical probabilities for red and black responses were 50% to 60%, for yellow responses 70% or above. The majority of significant effects was found in the left amygdala. Positive effects were found in both right and left LB and SF (c.f. Tab. 2), negative effects were localized in left LB, SF and CM. For visualization, probability maps were smoothed using a spatial filter with a 5 mm isometric Gaussian convolution kernel.

Both right and left amygdala showed significant auditory-stimulation-related BOLD effects (p<0.05, corrected for multiple comparisons, Fig. 3 and 4, Tab. 2). Positive signal changes were predominantly found in LB, negative signal changes were predominately observed in SF and CM. There were no significant voxelwise differences between consonant and dissonant musical stimuli.

**Table 2 pone-0000307-t002:** Significant music-related signal changes in LB, SF, and CM.

		Volume of Left Amygdala Responses (mm^3^)		Volume of Right Amygdala Responses (mm^3^)	
		Positive	Negative	Positive	Negative
**Subregion**	LB	60	23	13	-
	SF	25	60	2	-
	CM	-	57	-	-

Volumes with significant effects (in mm^3^) for the right and left amygdala presented separately for positive and negative signal changes. Results are given for the mean effect across all four music conditions (‘Mean’). LB: laterobasal group, SF: superficial group, CM: centromedial group.

**Figure 5:**
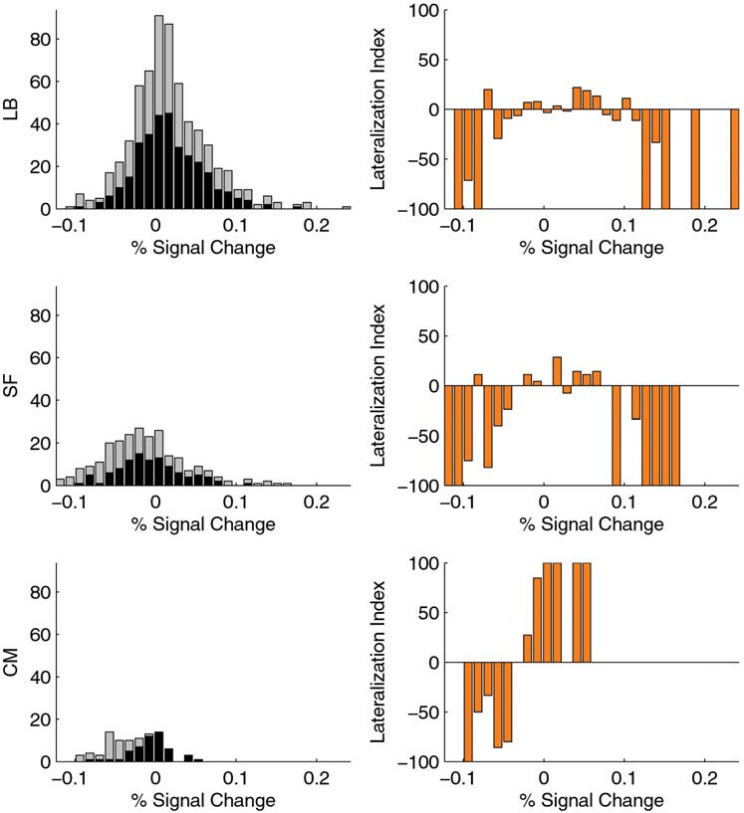
Lateralization patterns in the major subdivisions of the human amygdala. Histograms on the left show the distribution of BOLD percentage signal change (PSC) for voxels in: LB = laterobasal group, SF = superficial group, and CM = centromedial group. Values for the right (black) and left (light grey) amygdala are shown in a stacked way. For each PSC bin, a lateralization index (LI) was calculated. LIs of 100, −100, and 0 indicate purely right-sided, purely left-sided, and symmetrically distributed effects, respectively. LB and SF showed a similar lateralization pattern of both extremely positive and negative PSC predominating in the left amygdala. CM showed a different lateralization pattern with negative PSC predominating in the left and positive PSC in the right amygdala.

We investigated the lateralization patterns in LB, SF and CM by calculating a lateralization index (LI) as a function of PSC strength (Fig. 5). LB and SF showed a similar lateralization pattern with voxels with both the positive- and negative-most PSCs lateralized to the left amygdala. In contrast, CM showed a lateralization pattern with negative PSC predominating in the left and positive PSC in the right amygdala.

**Figure 6:**
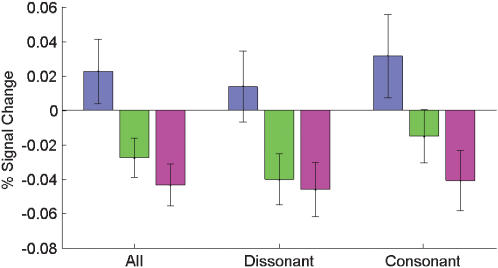
Response differences between amygdala subregions. We performed a region of interest (ROI) analysis to statistically evaluate response differences between the core regions (with 90–100% assignment probability) of the three amygdala subregions (see Material and Methods for further details). Bars in the above figure show the result of three averaging steps: first across all voxels of a given area, then either across all four music conditions, the dissonant conditions, or the consonant conditions, and finally across the 14 subjects. Results are given for left LB, SF, and CM in blue, green, and magenta. Error bars indicate the standard error of the mean from of the average across subjects. In each case, the differences between left LB and left SF and CM were significant at p<0.005. There were no corresponding significant differences in the right amygdala.

To determine whether there were significant differences in mean PSC between the amygdala subregions, we performed a region of interest (ROI) analysis. The ROI was defined as the area with 90% or 100% probability to belong to LB, SF, and CM. We determined the mean PSC for each right and left subregion for each individual subject. Based on this ROI data, we tested for differential responses in right and left amygdala subregions (Fig. 6). For the mean responses across all four music conditions, left LB showed significant higher PSC than left SF and left CM (p<0.005). Separate analysis of consonant/dissonant stimulus class revealed that the same difference between left LB and SF as well as left LB and CM was found for the consonant and for the dissonant stimuli (Fig. 6).

## Discussion

In the present study we used fMRI combined with probabilistic anatomical maps to investigate functional response properties of human amygdala subregions [26]. The utility of our approach critically rests on the EPI signal quality in the amygdala region. By application of a new online correction method for geometric EPI distortions based on the point spread function (PSF) [39] we achieved good EPI signal quality at 3 T in the amygdala region (Fig. 1). Our results clearly demonstrate the existence of significant fMRI response differences between probabilistically defined human amygdala subregions.

In the present study we used music as stimuli to evoke amygdala responses. Several previous functional imaging studies have probed the neural circuitry underlying music processing in a variety of functional contrasts: Using positron emission tomography (PET), Blood and colleagues investigated responses to melodies of varied degrees of dissonance [40], and Blood and Zatorre compared responses to subject-selected ‘favorite’ music minus music selected by the other subjects [14]. In another PET-study, Brown and colleagues [41] investigated music presentation versus rest. Based on fMRI, studies have compared unpleasant and pleasant music [15], music in minor and major mode [42], or music and scrambled music [43]. Only two of these imaging studies reported effects in the amygdala region [14], [15]. However, the specific response properties of the anatomical amygdala subregions were not assessed in these studies.

One of the previous studies reporting amygdala effects for musical stimuli focused on differential activation between pleasant and unpleasant pieces [15]. In contrast, other studies using for example gustatory, olfactory, or visual stimuli have emphasized the similarity of responses to both pleasant and unpleasant stimuli in the human amygdala [6], [7], [33], [34]. In line with these later results, the data of our study support the notion of a basically similar amygdala response pattern both to pleasant and to unpleasant stimuli (Fig. 3 and 6). In our study, this basic pattern was characterized by increased BOLD responses (‘activation’) in LB and decreased BOLD responses (‘deactivation’ [44]) in SF and CM. Beyond this basic pattern, further research is however needed to tease out the finer auditory-induced modulations of human amygdala responses along the valence and arousal dimensions (for a recent study in the olfactory domain, see ref. 8).

Importantly, there were no differences in familiarity ratings for both the pleasant and unpleasant tunes in our study. Subjects' familiarity with the experimental stimuli may shape amygdala responses, as several studies have shown [35]–[38] that the response of the human amygdala to emotional facial expressions diminishes with repeated presentations, that is, with increasing familiarity. The role of familiarity differences in previous studies of auditory-evoked human amygdala responses is less clear and could explain why amygdala-deactivation was found when subjects listened to highly familiar music in contrast to music selected by the other participants [14].

The basic response pattern within probabilistically defined human amygdala subregions found in our study fits well into general concepts of mammalian amygdala organization [21]. In various animal species LB contains auditory responsive neurons [45] and has been shown to be the main target of sensory, including auditory, afferents to the amygdala in anatomical studies [23]–[25]. The activation effects in LB that we observed may reflect a similar involvement of the human LB in the processing of auditory inputs. Further, reciprocal changes in firing probability between lateral and central medial amygdala neurons have been reported in cats [46]: Auditory stimulation caused firing probabilities of lateral and central medial neurons to oscillate in phase opposition, with an initial excitation in LB and inhibition in CM. It has been proposed that these reciprocal response patterns result from inhibitory influences of LB on CM, mediated by interposed intra-amygdala nuclei [46]. This reciprocal functional organization parallels the reciprocal auditory-evoked BOLD effects observed in the group mean effects of the current study for LB and CM. Whether BOLD signals in human LB and CM do show reciprocal behavior on a finer temporal scale remains to be investigated. Also in a previous fMRI study, reciprocal signal changes during implicit fear conditioning were reported for two spatially segregated responses in the amygdala region [47], suggesting that this reciprocal behavior may be a more general characteristic of the human amygdala.

Our results point also to different lateralization patterns for CM as compared with LB and SF. The general issue of lateralization of emotional functions in the human amygdala has been a matter of much debate [48], but lateralization in the context of subregional differences within the amygdala has not yet been considered. We found a predominance of the positive- and negative-most responses in LB and SF of the left amygdala. In contrast, the positive-most responses in CM predominated on the right and the negative-most responses on the left. This suggests that differentiating between LB, SF, and CM might be important for studies on lateralization of amygdala function. We used relatively unknown piano melodies of 24 second duration as auditory stimulation. Other studies have shown [36], [38] that the response-habituation process to emotional facial expressions is more rapid for the right than for the left amygdala. This could explain why we found, using relatively long stimuli, a predominance of the positive- and negative responses in the left amygdala (both in LB and SF). One might speculate whether shorter piano melodies evoke more bilateral amygdala activation in LB and SF.

The approach we have applied requires high BOLD imaging localization accuracy. Sources of spatial fMRI localization errors include EPI image distortions [39], draining vein effects [49], and inaccuracies arising from spatial data pre-processing [50]. We have addressed the problem of spatial EPI distortions, which are particularly prominent in the medial temporal lobe [51], by applying a new distortion correction method based on the BOLD point spread function [39]. Making use of the shift and broadening of the point spread function in phase encoding direction, the original signal location and intensity in phase encoding direction is recovered to result in an undistorted image [39]. While image distortions are more severe in the amygdala region as compared with other commonly examined brain regions, the draining vein problem can be assumed to be less pronounced in the amygdala for the following reasons: (1) the draining vein problem is more severe for large areas of activated neuronal tissue [49], but the amygdala, and especially its subnuclei, is/are relatively small. (2) Contamination by apparent activation along a draining vein will be most pronounced if a single vein drains the activated area (see also [49]). The amygdala is however drained by several veins [52], again reducing the draining vein problem; and, (3) these general considerations are confirmed by experimental data from BOLD venography. Venograms indicated that there are no sizable draining vein artifacts in the amygdala region [53].

Furthermore, the main findings of the present study were based on the anatomical core (with 90–100% assignment probabilities) of the amygdala subregions, increasing the robustness of our results against spatial errors and thus counteracting the potential effects as discussed above while still maintaining excellent anatomical specificity. These considerations and the fact that our present results conform to concepts of internal amygdala organization from animal research strengthen the assumption that the responses we have described truly reflect activation patterns of the major human amygdala subregions.

Our results imply that treating the human amygdala as a single entity, a predominant approach in current functional imaging studies, may be problematic. Averaging responses over voxels of the whole extent of the amygdala or restricting data interpretation to a single peak value may fail to capture the full complexity of amygdala responses. More importantly, averaging of positive and negative signals emanating from different amygdala subregions may even lead to false negative results as opposite effects may cancel each other out.

The present study is a first step in subdivision-level investigation of the human amygdala using probabilistic maps. To pursue this approach further, not only the spatial accuracy of amygdala fMRI needs to be optimized, but also a precise estimation of the magnitude of residual spatial localization errors in the amygdala region and of their impact on the functional results needs to be developed. In this way, the combination of fMRI with probabilistic anatomical maps may make an important contribution to our understanding of the internal functional organization of the human amygdala and of its neuronal mechanisms.
